# Influence of Manufacturing Conditions on Binder-Less Boards from Steam-Exploded Hemp Shives and Wheat Straw

**DOI:** 10.3390/ma15093141

**Published:** 2022-04-26

**Authors:** Ramunas Tupciauskas, Janis Rizhikovs, Martins Andzs, Oskars Bikovens

**Affiliations:** Latvian State Institute of Wood Chemistry, Dzerbenes 27, 1006 Riga, Latvia; janis.rizikovs@kki.lv (J.R.); martins.andzs@kki.lv (M.A.); oskars.bikovens@kki.lv (O.B.)

**Keywords:** hemp shives, wheat straw, steam explosion pre-treatment, response surface methodology, binder-less board, properties

## Abstract

In the current decade, based on the European Green Deal, new challenges of the wood-based panel industry have arisen, seeking for formaldehyde-free bio-based adhesives and broadening raw lignocellulosics. In order to contribute to the potential solution to the challenges, binder-less boards of steam-exploded (SE 220 °C/2 min) hemp shives and wheat straw were investigated. The objective of this study was to find out the optimal hot-pressing conditions in terms of temperature (150–200 °C) and time (5–16 min) for the boards with three density levels (800–1000–1200 kg·m^−3^). An experimental design was created and the influence of the variables on binder-less panels were evaluated using a randomized central composite design of the response surface methodology. Water absorption (WA) and thickness swelling (TS) during 24 h, modulus of elasticity (MOE), and modulus of rupture (MOR) in bending test, internal bonding (IB), and Fourier-transform infrared spectroscopy were determined for the obtained boards. Each detected physical-mechanical property of the obtained boards was described by statistical models being different at each density level. The optimal conditions of the obtained binder-less boards were different depending on the raw material and density. For example, the optimal conditions of the boards from SE wheat straw with a density of 800 kg m^−3^ were found at T = 220 °C and t = 15 min, with the achieved properties of WA = 53%, TS = 4%, MOE = 2750 N mm^−2^, MOR = 15.5 N mm^−2^, and IB = 0.64 N mm^−2^. Based on the achieved properties at the optimal conditions, the boards meet the requirements of the conventional particleboard Type P3 according to EN 312.

## 1. Introduction

The steam explosion (SE) technology is well-known since it was established in 1930 for wet process fiberboard production in the USA under the benchmark of Masonite [1]. After the rapid development of the wood-based panel industry (WBP), many types of synthetic adhesives, mostly containing formaldehyde, were integrated because of its technological advantages; one of the most important ones being fast curing of the adhesive and, therefore, the overall production time. Nowadays, taking more attention to the human life quality and environmental considerations, the WBP industry was challenged to reduce formaldehyde emission due to its recognized cancerogenic impact [2]. Another important challenge of the WBP industry is reducing raw wood materials due to the global shrinkage of forest area, high competition and high demand of WBP production [3]. At the same time, one of the European Commission’s six priorities for 2019–2024, the “European Green Deal”, highlights bioeconomy as the key element for innovative product lines based on renewable biological resources and encourages converting the waste streams of the production, as well as production residues and by-products, into value-added products [4].

SE is a rather simple process consisting of the application of saturated steam in a sealed reactor at elevated temperature (160–240 °C) and pressure (7–34 bar) during a relatively shorter time (up to 30 min). After the residence time exceeds, the reactor is rapidly opened and due to the decompression, the lignocellulosic material explodes splitting its physical and chemical structure to lower fractions [5]. This physical-chemical splitting results in degradation of hemicelluloses and lignin softening coating the fiber surface and further acting as self-adhesive components. Usually, SE process is characterized by a severity factor R0 which comprises the pre-treatment temperature and time [6]. The obvious positive effect of SE pre-treatment on the formation and properties of binder-less boards was investigated and proved for many biomasses, including wood and non-wood raw materials [7,8,9]. Therefore, the comeback of binder-less board production in the WBP industry, including non-wood raw materials, could be a valuable solution to the raised challenges.

Due to the steam expansion in the SE process, a lignocellulosic raw material is usually converted from the form of chips to the form of a substrate with a fibrous shape. The SE solid substrate contains inhomogeneous particles of different sizes, starting from only partially split chips to very small and single fibers depending on the regime and the device feature [5]. Therefore, SE substrates are needed to be homogenized to make a board composite with an even structure. Since it is not done by a classical defibration manner, the obtained board is intended to be called as binder-less particleboard and its properties are comparable to conventional particleboard and not fiberboard.

Industrial hemp (*Cannabis sativa* L.) has been approved for cultivation and production of many composites due to its strong bast fibers [10,11], becoming an expanding crop in Europe, including Latvia [12]. After the extraction of bast fibers, the hemp woody core, called shives or hurds, remains up to 75% of the stalk mass having a great potential to be used in particleboard production. The investigated particleboard from hemp shives bonded with synthetic PMDI resin conformed the ANSI requirements [13], while the binder-less board performed only from the shives by the thermoforming process showed very poor water resistance [14]. Some of our previous studies on the complex processing of industrial hemp shives have evaluated the production of furfural and self-binding panels [15] with an acoustic application [16].

The most abundantly used annual agricultural crop in Europe, including Latvia, is wheat (*Triticum aestivum*) with an approximately 2/3 standing area from all cereals. After grain harvesting, the straw is a residue with high potential to be used as bio-based composites. Different pre-treatment methods were reviewed to enhance the wheat straw application in building materials, the most effectively reported as steam cooking and steam explosion [17]. Investigation of wheat straw for the production of medium density fiberboards (MDF) with synthetic adhesives [18,19], inorganic-based particleboard [20] and binder-less board with enzymatic pre-treatment [21,22] has been demonstrated. Regarding binder-less boards from SE wheat straw, only one research paper was found, with the investigated SE temperature range of 160–170 °C [23].

Our preliminary studies regarding the utilization of SE hemp shives and wheat straw for binder-less board production have been intended to investigate the SE impact on the raw materials [24], optimal SE conditions and preliminary hot-pressing variables [25,26], and conditions for medium density boards with a higher thickness [27].

The novelty of the present study is the investigation of optimal hot-pressing conditions of binder-less boards made from SE hemp shives and wheat straw at three density levels (800 kg m^−3^, 1000 kg m^−3^ and 1200 kg m^−3^) based on our previously found optimal parameters [25,26] and the experimental design, including temperature (150–200 °C) and time (5–16 min). Based on the obtained experimental results, statistically predicted mathematical models were created to express each property of binder-less boards.

## 2. Materials and Methods

### 2.1. Raw Crops

Shives with a maximum of 2% of the remaining long fibers of the locally cultivated and available in Latvia retted industrial hemp variety Uso31 (Jelgava district, 56°38′54″ N 23°42′50″ E, SIA “NDRA”, Jelgava, Latvia) were used as the hemp raw material. Wheat straw was delivered from a farmer in the Limbaži district (57°31′ N 24°43′ E, ZS “TŪJASMUIŽA”, Zemnieki, Salacgrivas County, Latvia). Both raw materials were crushed separately in a knife mill (CM4000, LAARMANN, Roermond, The Netherlands) to pass a sieve with openings of Ø 10 mm, removing the fraction of ≤0.5 mm due to the high ash content [24].

### 2.2. SE Pre-Treatment

The crushed raw materials (0.5–10 mm) with a moisture content of ~10% were pre-treated separately in a steam explosion (SE) device of original construction with a 0.5 L batch reactor at the optimal conditions of temperature 220 °C and residence time of 2 min, (severity factor logR0 = 3.83) found in a previous study [25]. The SE reactor’s volume was filled by 90–95% of the raw material and the saturated steam injected within 3–5 s to reach the pressure of 23 bar and maintained for 2 min. After the end of the pre-treatment time, a ball valve was opened immediately and a rapid decompression resulted in a steam explosion, providing the pre-treated material to a receiver. Up to 25 batches were processed with the raw material in one cycle of the SE pre-treatment. The pre-treated crops were collected as received, oven-dried at a temperature of 60 °C to a moisture content of 2 ± 0.2%, and homogenized by crushing in a knife mill (Retsch SM100, Haan, Germany) to pass a 4 mm sieve. The moisture content and fiber fraction of the SE substrates were prepared based on a previous study [26].

### 2.3. Binder-Less Board Fabrication

The experimental design of the board fabrication was selected by a software of Design Expert 13 (Stat-Ease Inc., Minneapolis, MN, USA) using a full factorial central composite design coupled with the response surface methodology [28]. Two variables of pressing temperature (T) and time (t) were analyzed at three density levels: 800 kg m^−3^, 1000 kg m^−3^, and 1200 kg m^−3^ (Table 1).

A total of eleven experimental trials were performed per one density level: four factorial trials, four axial trials, and three center point replicates (experiments 9–11). The range of the board pressing temperature and time for the boards with the density of 1200 kg m^−3^ was selected differently based on a previous study [26].

**Figure 1:**
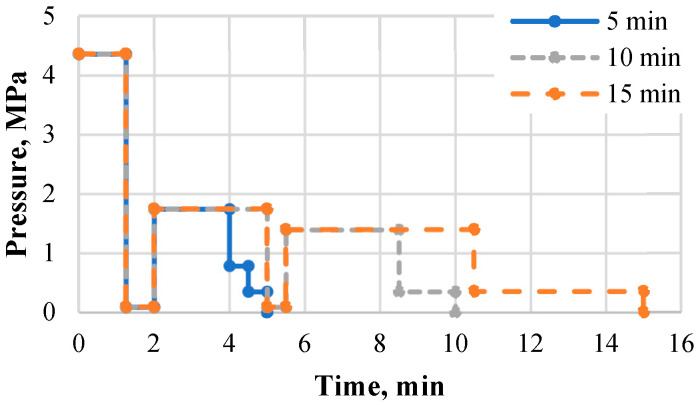
Time–pressure diagram for binder-less board fabrication.

One-layer binder-less boards with a thickness of 6 mm were hot-pressed in a hydraulic hot-press (Joos LAP 40, Ulm, Germany). At the beginning, the prepared substrate (131.3 g, 164.1 g and 195.7 g for the densities of 800 kg m^−3^, 1000 kg m^−3^ and 1200 kg m^−3^, respectively) was placed in a mold with inner dimensions of 150 × 180 mm and pre-pressed under ~1.3 MPa for 15 s to form a mat. Then, the mold was taken off and the pressing cycle started. Two board samples were fabricated per pressing cycle. The time-pressure diagram included the maximum pressure (depending on a board density, Table 1) during the first 75 s, followed by one breathing for 45 s for all boards and two breathings with a 3 min interval for the boards with the pressing times ≥10 min, followed by decreasing pressure down to 0.3 MPa before the opening (Figure 1). To ensure the highest density level (1200 kg m^−3^), the maximum pressure at the beginning of the pressing cycle was needed to be higher than for the boards with a lower density. To provide constant board thickness and set density, thickness limiting bars were used at each pressing plate corner.

The obtained boards from pre-treated Uso hemp shives (Uso-board) and wheat straw (Wh-board) were conditioned at a relative humidity of 65 ± 5% and the temperature of 20 ± 2 °C to achieve a constant mass before the board testing [29,30,31].

### 2.4. Binder-Less Board Evaluation

The obtained boards were evaluated by the modulus of elasticity (MOE) and the modulus of rupture (MOR) in the three-point bending test [29], internal bonding (IB) determining the tensile strength perpendicular to the plane of the board [31], and thickness swelling (TS) and water absorption (WA) after the specimens immersion in water for 24 h [30]. Mechanical tests (MOE, MOR, IB) were performed on a ZWICK/Z100 (Ulm, Germany) universal machine for testing the resistance of materials. Six specimens per board type were determined in each test to calculate the average value of each property.

The factors of the influence on the mean values of the tested properties were analyzed by Excel software using the one-way ANOVA tool at the significance level α = 0.05 [28].

Fourier transform infrared (FTIR) spectra of raw and pretreated crops and the obtained boards were recorded in KBr (IR grade, Sigma Aldrich, Darmstadt, Germany) pellets by a Thermo Fisher Nicolet iS50 spectrometer (Waltham, MA, USA) in the range of 4000–700 cm^−1^ with the resolution of 4 cm^−1^ and the number of scans 32. All spectra were normalized to the highest absorption maxima in the range of 2000–700 cm^−1^.

## 3. Results and Discussion

The results of the performed experimental design (Table 1) were analyzed by the data processing software Design Expert 13. The influence of the experimental design variables on each board property was described by the software proposed models for each density level separately.

### 3.1. Binder-Less Fiberboards with a Density of 800 kg m^−3^

#### 3.1.1. Density and Internal Bonding

**Figure 2:**
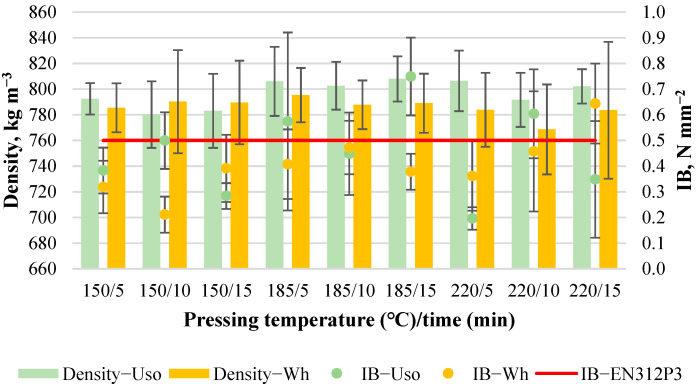
Density and IB changes of binder-less boards with a density of 800 kg m^−3^ depending on the crop and pressing variables.

The obtained boards with a density of 800 kg m^−3^ from both raw materials were of good quality and without any observed cracks within the board thickness profile. The boards’ density and IB values depending on pressing variables are shown in Figure 2. The average density of Uso- and Wh-800 boards slightly varies from 780 to 806 kg m^−3^ and from 769 to 795 kg m^−3^, respectively. The average IB value of Uso- and Wh-800 boards varies significantly from 0.2 to 0.75 N mm^−2^ and from 0.21 to 0.64 N mm^−2^, respectively (Figure 2).

The high standard deviation of the detected IB values indicates the inhomogeneous strength across the board area. Generally, a higher IB value was detected in the middle specimen of a board sample while lower IB values were obtained in the specimens cut from the outer edges of the board samples. Such a result could be related to the edge effects of the rather small board samples (150 × 180 mm) caused by the inherent temperature and pressure variations experienced by particles in the outer edges of a mattress [32]. A higher IB value was achieved at 185/15 by Uso-board (0.75 N mm^−2^) and at 220/15 by Wh-board (0.64 N mm^−2^), indicating the best conditions of board bonding strength formation depending on the crop. It should be noted that the obtained higher IB values of Uso-800 boards, including at 185/5 and 220/10, and the Wh-800 board at 220/15 exceed the requirement of conventional particleboards (≥0.5 N mm^−2^, Figure 2) applied in a humid condition proposed by the standard EN 312 P3 [33].

At the same time, the highest IB value of the Wh-800 board obtained in this study exceeds more than twice the IB value (0.26 N mm^−2^) of the Wh-board obtained from SE wheat straw at 170 °C for 10 min (logR0 = 3.06) and hot-pressed at 190 °C for 8 min [23]. This and many other cases prove that both SE and hot-pressing conditions are very essential in IB strength formation, producing binder-less board from the SE lignocellulosic material [7,8]. It should be noted that the IB value of conventional MDF boards produced from wheat straw with synthetic melamine-urea-formaldehyde (MUF) adhesives [18] was close enough to the highest IB value of Wh-800 board achieved within this study (0.67 N mm^−2^ vs. 0.64 N mm^−2^, respectively).

Analyzing the obtained IB values, no clear tendency depending on variables is observed (Figure 2). Looking for a mathematical expression of the obtained boards properties within the used factorial design ranges, Design Expert software was applied. A fit summary of the suggested models [28] for each property of the boards with the density of 800 kg m^−3^ is shown in Table 2. No mathematical model was suggested to express the IB variation of Uso-800 boards based on the obtained actual values within the pressing variables. Therefore, in this case, only the overall mean with a value of 0.45 N mm^−2^ could be used to predict within the factorial design space.

In the case of Wh-800 boards, a linear regression model was suggested to express the IB variation Equation (1); however, it was insignificant, including both factors, as can be seen from the obtained statistical terms summarized in Table 2. Despite the insignificance of the model proposed in Equation (1), it can be used to navigate the design space according to the calculated adequate precision which measures the signal to the noise ratio being greater than 4.

IB_Wh800_ = 0.003(T) + 0.011(t) − 0.169 (1)

#### 3.1.2. Board Bending Properties

**Figure 3:**
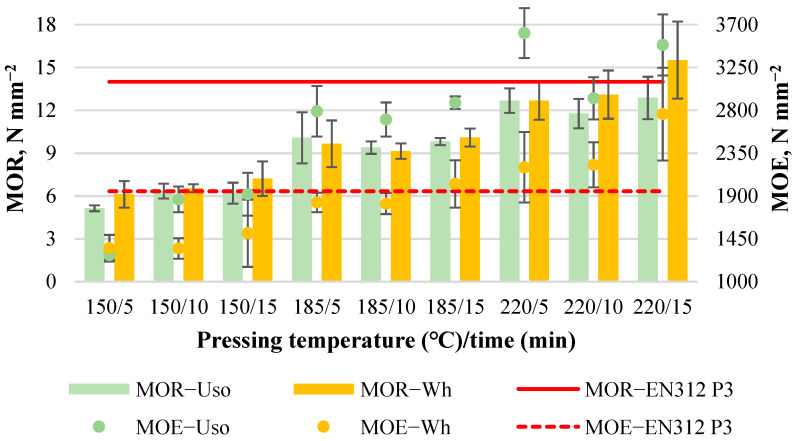
Bending properties of binder-less boards with a density of 800 kg m^−3^ depending on the crop and pressing variables.

The average MOR value increases significantly with increasing pressing temperature, from 5.1 to 12.9 N mm^−2^ and from 6.1 to 15.5 N mm^−2^ for Uso- and Wh-800 boards, respectively (Figure 3). Similarly, the average MOE value increases significantly with increasing pressing temperature, from 1280 to 3612 N mm^−2^ and from 1350 to 2760 N mm^−2^ for Uso- and Wh-boards, respectively (Figure 3). An increase in bending properties with the increase in pressing temperature was observed by other authors as well [14,34] indicating the optimal condition for the formation of inter-fiber bonds resulting to a maximum board mechanical strength. Under the optimal condition which may differ for different lignocellulosics occurs some significant chemical interactions including (1) lignin condensation reaction, (2) crosslinking between lignin and polysaccharides, and (3) the formation of covalent bonds between the constituents of lignocellulosic polymers [8].

It is worth noting that a significantly higher MOR value is achieved for Wh-board; however, a significantly higher MOE value is achieved for Uso-board. This indicates the structural differences observed between the raw materials on the micro scale [25], pre-treated Uso hemp shives being more elastic than wheat straw. Differences in the board properties between the used crops could be attributed to the differences in chemical composition of the crops. As was reported before [24], cellulose content of SE-220/2 wheat straw and Uso hemp shives was the same (40.00 ± 0.14%); however, lignin and hemicelluloses contents were different (28.7%/12.5% and 33.1%/13.3%, respectively). Furthermore, cellulose degree of polymerization of these samples also differs significantly: 291 ± 2 for Uso and 407 ± 2 for wheat straw [25].

It should be also noted that the obtained higher value of MOR in the case of the Wh-board at 220/15 and the MOE values of Uso-boards achieved at T ≥ 185 °C and Wh-boards at T = 220 °C meet the standard EN 312 P3 value requirements (Figure 3) These achievements indicate a high potential for binder-less boards, which could be used as a building material for structural applications.

It is worth noting that the MOR value of the binder-less board from wheat straw obtained in a similar study was unbelievably higher (19.8 N mm^−2^), taking into account the lower SE severity (3.06) and pressing conditions at 190/8 [23]. The MOR value of MDF obtained from wheat straw was even higher, 26.8 N mm^−2^, resulted by the defibration process and the addition of synthetic MUF adhesive [18].

The MOR variation depending on the variables was expressed by significant linear models for Uso- and Wh-800 boards proposed in Equations (2) and (3), respectively. Because of the approved significance of the factor A (Table 2), the obtained equations can be interpreted as follows: increasing the T by 1 °C, the MOR value will increase by 0.09 N mm^−2^ and by 0.1 N mm^−2^ for Uso- and Wh-boards, respectively:

MOR_Uso800_ = 0.09(T) − 0.03(t) − 8.25 (2)

MOR_Wh800_ = 0.10(T) + 0.15(t) − 10.48 (3)

The MOE variation depending on the variables was expressed by the significant linear model (4) for the Uso-board and by the significant quadratic model (5) for the Wh-board:

MOE_Uso800_ = 23.62(T) − 20.18(t) − 1947.45 (4)

MOE_Wh800_ = 470.84 + 8.76(T) − 187.28(t) +0.57(T t) − 0.0008(T^2^) + 5.58(t^2^) (5)

In the case of the Uso-board, the MOE variation is significantly dependent on the factor A: increasing T by 1 °C, the MOE value will increase by 23.62 N mm^−2^. In the case of the Wh-board, the MOE variation is significantly dependent on both factors A and B, including its interaction and quadratic level except for B (Table 2).

#### 3.1.3. Water Resistance Properties

**Figure 4:**
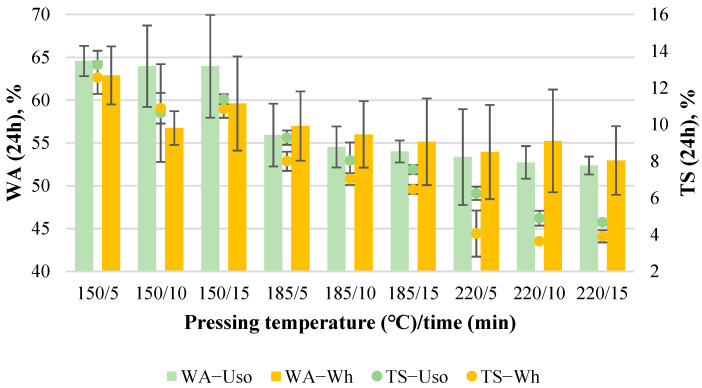
WA and TS of binder-less boards with a density of 800 kg m^−3^ depending on the crop and pressing variables.

The average WA value of the obtained binder-less boards decreases significantly with increasing pressing temperature, from 65% to 52% and from 63% to 53% for Uso- and Wh-800 boards, respectively (Figure 4). The decrease of the WA value of binder-less boards from SE wheat straw depending on hot-pressing time was demonstrated by another study where the lowest WA value was reported as 61.5% [23]. It is well-known that the WA of particleboards or fiberboards is highly dependent on the board density—the higher the density, the lower WA is achieved due to the higher compaction of particles [32]. This study reveals that WA is also significantly dependent on pressing temperature that was confirmed by the obtained statistical models (6) and (7), the significance of which was approved by ANOVA summarized in Table 2.

In the case of Uso-boards, the WA variation was expressed by the quadratic model with both significant factors:

WA_Uso800_ = 189.34 − 1.27(T) − 0.27(t) − 0.0005(T t) + 0.003(T^2^) + 0.01(t^2^) (6)

In the case of Wh-boards, the WA variation was described by the significant linear model (7): increasing T by 1 °C and t by 1 min, WA will decrease by 0.08% and 0.2%, respectively.

WA_Wh800_ = 73.59 − 0.08(T) − 0.203(t) (7)

The average TS value of the boards also decreases significantly with increasing both pressing variables, from 13% to 4%, very similarly for both Uso- and Wh-800 boards (Figure 4). Slightly lower TS values are observed for Wh-boards with a significant difference in some cases (e.g., at 185/5 and 220/5, Figure 4). It should be noted that the obtained TS values of both Uso- and Wh-boards in all experimental cases perfectly meet the standard EN 312 P3 value requirement, which is ≤20%. It has been reported [18] that the MDF from wheat straw with MUF adhesives contains TS values in the range of 7.1–7.8%, which fit with the TS values obtained within this study, starting at 185/5 and higher (Figure 4). These results again indicate the high potential of the binder-less boards, which could be used as a building material for structural applications even in humid conditions.

The dependence of the boards’ TS on the variables significantly describes the linear (8) and quadratic (9) models for Uso- and Wh-boards, respectively:

TS_Uso800_ = 27.21 − 0.09(T) − 0.17(t) (8)

TS_Wh800_ = 45.897 − 0.261(T) − 0.847(t) + 0.002(T t) + 0.0003(T^2^) + 0.016(t^2^) (9)

Both factors were found to be significant, describing the TS variation by the models (Table 2). In the case of Uso-boards, Equation (8) can be interpreted as follows: increasing T by 1 °C and t by 1 min, TS will decrease by 0.09% and 0.17%, respectively.

#### 3.1.4. Model Fit

**Figure 5:**
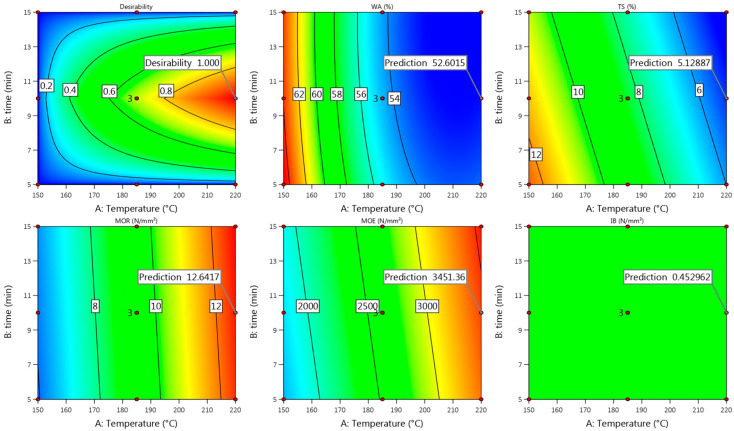
Contour plots with predicted values of Uso-800 binder-less board properties at T = 220 °C and t = 10 min.

**Figure 6:**
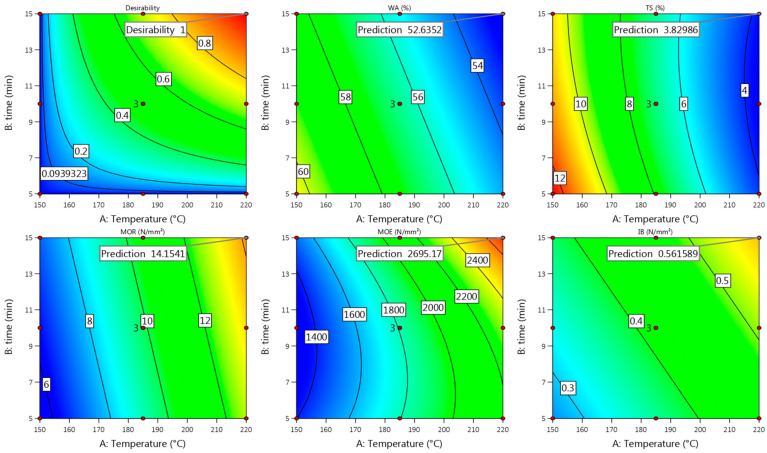
Contour plots with predicted values of the Wh-800 binder-less board properties at T = 220 °C and t = 15 min.

Summarizing all the obtained properties of the Uso-800 board, the best actual values were achieved at the pressing temperature of 220 °C and time of 10 min (Figure 2, Figure 3 and Figure 4) which were found to fit well with the predicted ones (Figure 5) according to Equations (2), (4), (6) and (8) except IB, as mentioned in the Section 3.1.1. The best actual values of the obtained Wh-800 board properties were achieved at the pressing temperature of 220 °C and time of 15 min (Figure 2, Figure 3 and Figure 4), which also were found to fit well with the predicted ones (Figure 6) according to Equations (1), (3), (5), (7) and (9) without exceptions. The calculated difference between the actual and predicted values of the board properties (except IB in the case of Uso-800) from both crops is within the confidence level of 95%.

### 3.2. Binder-Less Fiberboards with a Density of 1000 kg m^−3^

#### 3.2.1. Density and Internal Bonding

Generally, the obtained boards with a density of 1000 kg m^−3^ were of good quality except for the Wh-boards hot-pressed at 185/5, 220/5, and 220/10—these contained blisters or cracks within the board thickness or area profile. This revealed certain circumstances unsuitable for binder-less board production from pre-treated wheat straw. This phenomenon also indicates and reveals a significant dependence of hot-pressing conditions on the board density. As was mentioned in the previous subsection, the Wh-800 boards obtained at these conditions were of good quality. Despite the cracks which were generally formed in the board center, it was possible to cut some specimens for the evaluation of the board’s properties.

**Figure 7:**
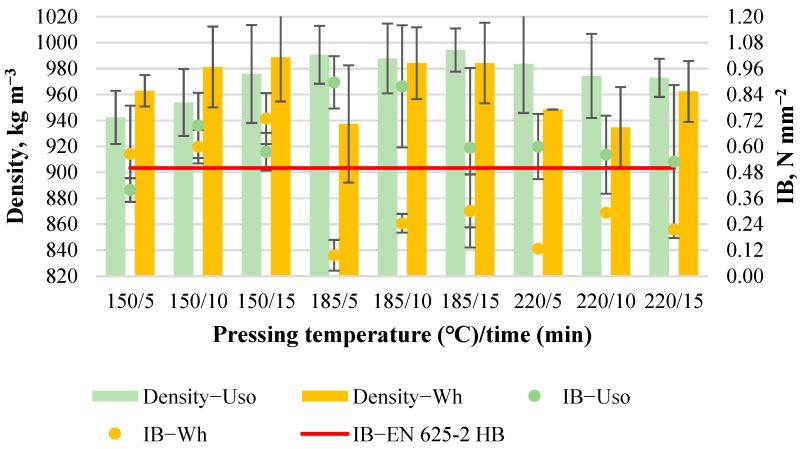
Density and IB of binder-less boards with a density of 1000 kg m^−3^ depending on the crop and pressing variables.

The average density of the obtained Uso- and Wh-1000 boards significantly varies from 942 to 994 kg m^−3^ and from 935 to 990 kg m^−3^, respectively (Figure 7). The achieved lower density for the Uso-board at 150/5 is related to the spring back effect that was proved by a higher thickness (5.97 vs. 5.77 mm, in average, for Uso-1000 boards). This indicates too low pressing temperature and time under which it is not possible to obtain a stable composite from pre-treated Uso hemp shives. The density of Uso-boards increases with increasing pressing time at 150 °C and slightly decreases at 220 °C. A lower density was detected also for Wh-boards (at 185/5, 220/5 and 220/10) with achieved blisters/cracks, possibly due to the loss of volatile compounds which were formed from unreacted compounds during the hot-pressing [35].

The average IB value of the boards with a density of 1000 kg m^−3^ varies significantly from 0.40 to 0.90 N mm^−2^ and from 0.10 to 0.73 N mm^−2^ for Uso- and Wh-boards, respectively (Figure 7). Comparing the overall average IB value (obtained from 11 experiments, Table 1) of Uso-800 and Uso-1000 boards, it was increased from 0.45 N mm^−2^ to 0.64 N mm^−2^. The increase of the IB value indicates a significant impact of the board density. However, in the case of Wh-1000 boards, the increase of the overall average IB value did not occur, while the maximum—0.73 N mm^−2^—was exceeded at 150/15. At the same time, the maximum IB value of the Uso-1000 board—0.90 N mm^−2^—was achieved at 185/5, which exceeded the maximum IB value of Uso-800 (0.75 N mm^−2^ at 185/15) and Wh-1000 boards (Figure 2 and Figure 7). The lower IB values of the Wh-1000 boards achieved at 185/5 (0.1 N mm^−2^) and 220/5 (0.13 N mm^−2^) were impacted by the formed blisters/cracks, meaning too low pressing time. However, all other samples obtained at T ≥ 185 °C also achieved low IB values compared to Uso-boards (Figure 7), indicating significant structural differences of the crops.

To describe the IB variation of Uso-1000 boards depending on *T* and *t*, the quadratic model was suggested as expressed in Equation (10), although it is not significant (Table 3). The calculated adequate precision of the model was lower than 4, therefore, the overall mean (0.64 N mm^−2^) may be a better predictor, similarly to the case of Uso-800 boards.

IB_Uso1000_ = 0.07(T) + 0.15(t) − 0.0003(T t) − 0.0002(T^2^) − 0.005(t^2^) − 6.72 (10)

Differently, similar to the case of Uso-boards, the suggested quadratic model (11) to describe the IB variation of Wh-1000 boards is significant, including both variable factors as shown in Table 3:

IB_Wh1000_ = 6.59 − 0.07(T) + 0.07(t) − 0.0001 (T t) + 0.0002(T^2^) − 0.002(t^2^) (11)

#### 3.2.2. Board Bending Properties

**Figure 8:**
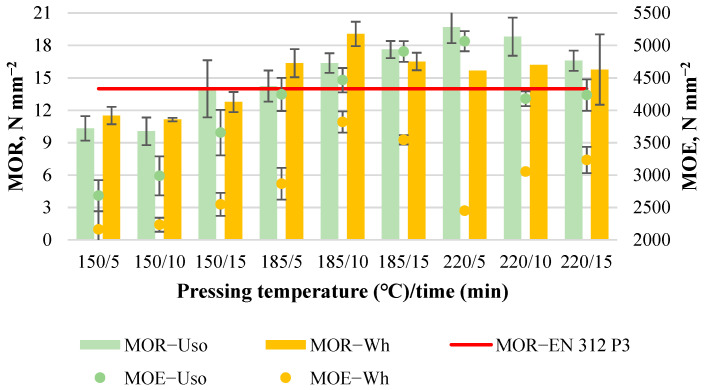
Bending properties of binder-less boards with a density of 1000 kg m^−3^ depending on the crop and pressing variables.

The average MOR value increases significantly with increasing T, from 10.1 to 19.7 N mm^−2^ and from 11.1 to 19.1 N mm^−2^ for Uso- and Wh-1000 boards, respectively (Figure 8). The average MOE value of the boards also increases significantly with increasing T, from 2685 to 5065 N mm^−2^ and from 2160 to 3820 N mm^−2^ for Uso- and Wh-boards, respectively (Figure 8). The maximum MOR value of the Uso-1000 board (19.7 N mm^−2^) was achieved at 220/5, which was almost equal to the maximum of the Wh-1000 board (19.1 N mm^−2^) achieved at 185/10. Considering the increase of density from 800 to 1000 kg m^−3^ (~25%), the maximum MOR value was increased by 53% and 23% for Uso- and Wh-boards, respectively. Differently from MOR, the MOE values of Uso-1000 boards are significantly higher compared to those of Wh-1000 boards (Figure 8) as in the case of a lower density (Figure 3), and the maximum was increased also by 40% and 38%, respectively.

Based on the obtained results, the MOR variation depending on the variables was expressed by the significant two factor interaction (2FI) model for the Uso-1000 board in Equation (12) and the quadratic model for the Wh-1000 board in Equation (13). In both regressions, only the factor T and its interaction with t in Equation (12) are statistically significant (Table 3).

MOR_Uso1000_ = 0.19(T) + 1.92(t) − 0.009(T t) − 21.97 (12)

MOR_Wh1000_ = 1.24(T) + 1.24(t) − 0.001(T t) − 0.003(T^2^) − 0.04(t^2^) − 108.48 (13)

The MOE variation depending on the variables was expressed by significant quadratic models for Uso- and Wh-1000 boards in Equations (14) and (15), respectively:

MOE_Uso1000_ = 277.53(T) + 323.94(t) − 2.57(T t) − 0.63(T^2^) + 8.96(t^2^) − 24832.6 (14)

MOE_Wh1000_ = 267.68(T) + 208.59(t) + 0.56(T t) − 0.71(T^2^) − 12.58(t^2^) − 23200.8 (15)

In the case of Uso-1000 board, the MOE variation is significantly dependent on factor T and its interaction with t. In the case of the Wh-1000 board, the MOE variation is significantly dependent on both variable factors (Table 3).

#### 3.2.3. Water Resistance Properties

**Figure 9:**
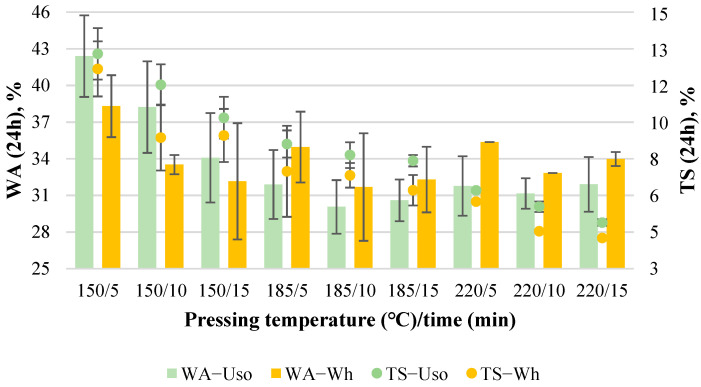
WA and TS of binder-less boards with a density of 1000 kg m^−3^ depending on the crop and pressing variables.

The average WA value of the boards varies significantly from 30% to 42% and from 32% to 38% for Uso-1000 and Wh-1000 boards, respectively (Figure 9). The increase of density from 800 to 1000 kg m^−3^ resulted in a decrease of the WA value by 43% and 40% for Uso- and Wh-1000 boards, respectively.

At the density level of 1000 kg m^−3^, WA was found to be significantly dependent on both variables that was approved by the obtained significant quadratic models. In the case of Uso-boards, almost all model terms are significant except for t^2^:

WA_Uso1000_ = 193.32 − 1.48(T) − 3.145(t) − 0.01(T t) + 0.003(T^2^) + 0.03(t^2^) (16)

In the case of Wh-boards, only T is not significant that was approved by ANOVA as summarized in Table 3:

WA_Wh1000_ = 95.83 − 0.5(T) − 3.08(t) + 0.007 (T t) + 0.001(T^2^) + 0.07(t^2^) (17)

The average TS value decreases significantly with increasing both pressing variables, from 13% to 5% and from 12% to 4% for Uso-1000 and Wh-1000 boards, respectively (Figure 9). The increase of density from 800 to 1000 kg m^−3^ did not promote the improvement of TS for both Uso- and Wh-boards. The significant linear models (18) and (19) were suggested to describe the dependence of TS on the variables for both Uso- and Wh-boards:

TS_Uso1000_ = 25.29 − 0.08(T) − 0.176(t) (18)

TS_Wh1000_ = 22.93 − 0.07(T) − 0.19(t) (19)

Both variable factors were found to be significant (Table 3), describing the TS variation by the models (18) and (19). Therefore, in the case of Uso-boards, increasing T by 1 °C and t by 1 min, TS will decrease by 0.08% and 0.176%, respectively. In turn, in the case of Wh-boards, increasing T by 1 °C and t by 1 min, TS will decrease by 0.07% and 0.19%, respectively.

#### 3.2.4. Model Fit

**Figure 10:**
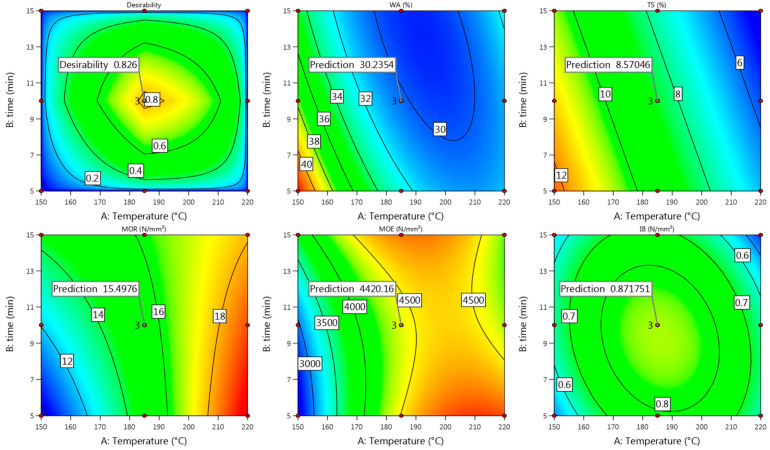
Contour plots with predicted values of Uso-1000 binder-less board properties at T = 185 °C and t = 10 min.

Summarizing all the obtained properties of Uso-1000 boards, good enough actual values were achieved at the pressing temperature of 185 °C and time of 10 min (Figure 7, Figure 8 and Figure 9), which fit well with the predicted ones (Figure 10) according to the above presented Equations (10), (12), (14), (16) and (18). The difference between the actual and predicted values is within the confidence level of 95%, meaning that the obtained quadratic model (10) for IB could be used as well at least at these conditions. Considering the obtained and predicted results, it could be concluded that the optimal conditions for production of Uso-1000 board with a thickness of 6 mm are 210/10. The conditions were approved by an additional experimental trial and the following results were obtained: D = 952 kg m^−3^, WA = 37%, TS = 8%, MOR = 18.6 N mm^−2^, MOE = 4910 N mm^−2^, IB = 1.24 N mm^−2^. The obtained results fully meet the requirements of the standard EN 312 P3.

**Figure 11:**
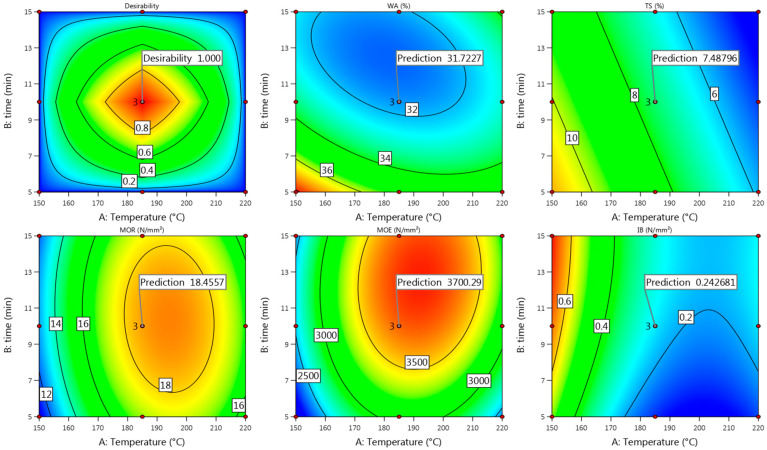
Contour plots with predicted values of Wh-1000 binder-less board properties at T = 185 °C and t = 10 min.

Comparing the achieved actual values of the Wh-1000 board properties obtained at the pressing temperature of 185 °C and time of 10 min (Figure 2, Figure 3 and Figure 4) with the predicted ones (Figure 11), it could be noted that these fit well, proving the suitability of the proposed Equations (11), (13), (15), (17) and (19). Based on the obtained and predicted results, it could be concluded that the optimal condition for production of Wh-1000 board with a thickness of 6 mm is 185/10. The condition was approved by a repeated experimental trial and the obtained results were the same even with the improved IB value of ≥0.8 N mm^−2^. The improvement resulted from the increased MC (≥15%) of the raw wheat straw before SE. The obtained results fully meet the requirements of the standard EN 312 P3, making the boards suitable for application in humid conditions.

### 3.3. Binder-Less Fiberboards with a Density of 1200 kg m^−3^

#### 3.3.1. Density and Internal Bonding

Implementing the experimental design of the boards with a density of 1200 kg m^−3^ (Table 1), plenty of failures in terms of the formed blisters or cracks within the board thickness or area profile were revealed. Uso-1200 boards made at T = 175 °C (except at t = 16 min) contained blisters within the board thickness profile in the middle area of the board sample. Wh-1200 boards made at T ≥ 165 °C contained both blisters and cracks within the board thickness or area profile. This revealed the unsuitable pressing temperatures for high-density binder-less board production from SE crops. The phenomenon is related to the difficult vapor-gas diffusion within the board mat during hot-pressing of highly compressed particles [32]. The pressing temperature resulting in the board formation differences depending on the raw material could be attributed to the obtained different thermal properties. For example, the glass transition region was detected in the range of 163–192 °C and 135–137 °C for SE-220/2 Uso hemp shives and wheat straw samples, respectively [24]. As in the case of the boards with the density of 1000 kg m^−3^, it was possible to cut some specimens for the evaluation of the board’s properties.

**Figure 12:**
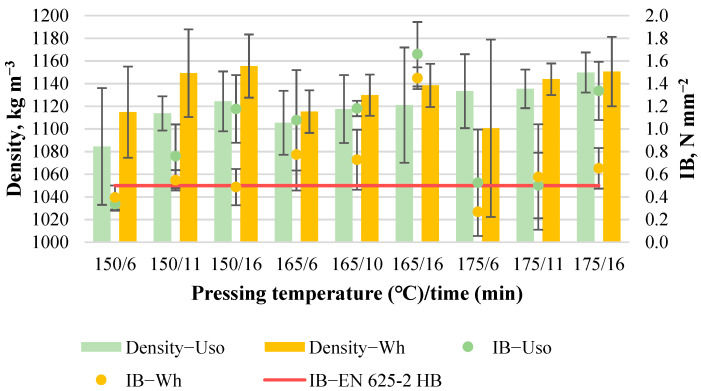
Density and IB of binder-less boards with a density of 1200 kg m^−3^ depending on the crop and pressing variables.

The average density of Uso- and Wh-1200 boards varies from 1085 to 1150 kg m^−3^ and from 1101 to 1151 kg m^−3^, respectively (Figure 12). One of the reasons for the unreached target density (1200 kg m^−3^) is a spring back effect that was proved by a higher thickness compared to the case of the boards with a lower density (1000 kg m^−3^)—5.92 mm and 5.77 mm, respectively. The lower density is observed for the boards obtained at shorter times. Another reason for the lower density is the formed blisters and cracks through which volatile organic compounds vapored rapidly immediately after the hot-pressing.

The average IB value varies significantly from 0.34 to 1.66 N mm^−2^ and from 0.27 to 1.45 N mm^−2^ for Uso- and Wh-1200 boards, respectively (Figure 12). The rapid maximum of the IB value was increased by 85% and 99% for Uso- and Wh-board, respectively, due to the density increase from 1000 to 1200 kg m^−3^. The highest IB values of the obtained boards were achieved at 165/16 from both pre-treated crops. The best result of IB for Wh-1200 board was not anticipated due to the observed blister at 165/16. However, the blister was located at the center of the board sample and the specimens cut for the board evaluation did not contain the inner gaps. This indicates that an optimal solution for Wh-1200 board production without blisters could be achieved at lower T or higher t. The lowest achieved IB values were related to the low temperature (150 °C) and the formed blisters/cracks.

The IB of the Uso-1200 board depending on the pressing variables was suggested to be expressed by the significant quadratic regression with the initially predicted R^2^ = 0.3376 and two significant factor terms (Table 4). The insignificant model terms such as (T t) and (t^2^) were removed that resulted in an increase of the predicted R^2^ = 0.5851 and the decreased coded *p*-value = 0.0079:

IB_Uso1200_ = 1.17(T) + 0.07(t) − 0.004(T^2^) − 94.47 (20)

The IB of Wh-1200 board depending on the pressing variables was also expressed by the suggested quadratic regression. However, the model was statistically insignificant and with a negative predicted R^2^ value (Table 4). Therefore, insignificant terms such as (T t) and (t^2^) were excluded from the model, resulting in the reduced quadratic model with a decreased *p*-value = 0.0693:

IB_Wh1200_ = 0.91(T) + 0.04(t) − 0.003(T^2^) − 73.65 (21)

#### 3.3.2. Board Bending Properties

**Figure 13:**
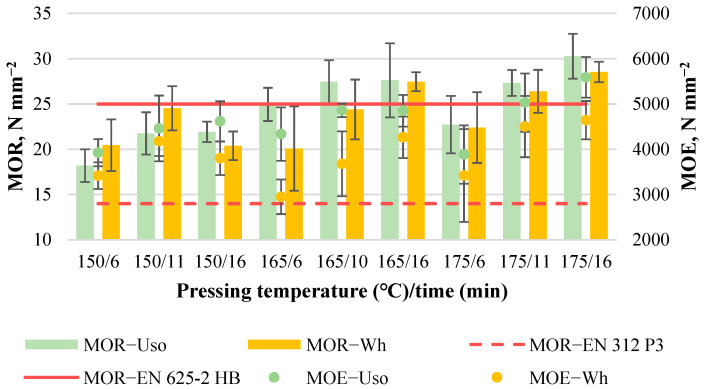
Bending properties of binder-less boards with a density of 1200 kg m^−3^ depending on the crop and pressing variables.

The average MOR value of Uso- and Wh-1200 boards increases significantly with increasing pressing temperature and time, from 18.2 to 30.3 N mm^−2^ and from 20.1 to 28.5 N mm^−2^, respectively (Figure 13). The results of the lowest bending properties were affected by the formed inner gaps of the obtained board samples. The lower values of all mechanical properties were observed for the middle specimens of the board samples. At the same time, the board samples obtained without inner gaps demonstrated a bit higher value of the mechanical properties for the middle specimens, meaning favorable pressing conditions. The highest MOR/MOE values were achieved at 175/16 for both Uso- and Wh-boards despite the presence of blisters in the case of the Wh-board. The conditions at 165/10 (only for the Uso-1200 board), 165/16, and 175/11 also promoted the high MOR/MOE values; however, those are not suitable for Wh-boards because of the formation of blisters. The increase of density from 1000 to 1200 kg m^−3^ resulted in an increase of the maximum MOR value by 54% and 50% for Uso- and Wh-boards, respectively.

The MOR of the Uso-1200 board depending on the pressing variables was suggested to be expressed by the significant quadratic regression with three significant model terms (Table 4):

MOR_Uso1200_ = 6.08(T) − 0.37(t) + 0.01(T t) − 6.87(T^2^) − 0.06(t^2^) − 486.54 (22)

The MOR of Wh-1200 board was suggested to be expressed by the significant linear regression (23) with both significant variable factors (Table 4). This means that increasing T by 1 °C and t by 1 min, MOR will increase by 0.16 N mm^−2^ and 0.41 N mm^−2^, respectively:

MOR_Wh1200_ = 0.16(T) + 0.41(t) − 6.87 (23)

The average MOE value of Uso- and Wh-1200 boards increases significantly with increasing pressing temperature, from 3880 to 5590 N mm^−2^ and from 3400 to 4640 N mm^−2^, respectively (Figure 13). The tendency of MOE dynamics depending on the pressing conditions is very close to the MOR dynamics presented above. The increase of density from 1000 to 1200 kg m^−3^ resulted in an increase of the maximum MOE value by 10% and 22% for Uso- and Wh-boards, respectively.

The MOE of the Uso-1200 board was suggested to be expressed by the significant linear regression (24) with one significant model term (Table 4). This means that increasing t by 1 min, MOE will increase by 93.81 N mm^−2^:

MOE_Uso1200_ = 162.15 + 21.39(T) + 93.81(t) (24)

The MOE of the Wh-1200 board was suggested to be expressed also by the linear regression (25), the fit summary of which is not so good; however, the pressing time was found to be as the significant factor (Table 4). Despite the insignificance of the model, it can be used to navigate the design space according to the calculated adequate precision being greater than 4. This means that increasing t by 1 min, MOE will increase approximately by 100 N mm^−2^:

MOE_Wh1200_ = 586 + 13.37(T) +99.94(t) (25)

#### 3.3.3. Water Resistance Properties

**Figure 14:**
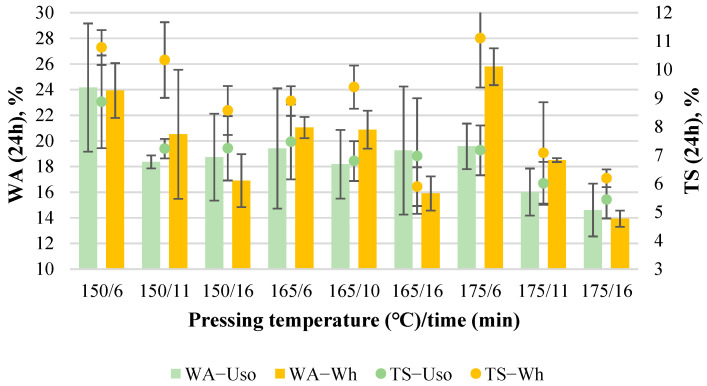
WA and TS of binder-less boards with a density of 1200 kg m^−3^ depending on the crop and pressing variables.

The average WA value of Uso- and Wh-1200 boards decreases with increasing variables similarly, from 24% to ~15% (Figure 14). The highest WA value of the Wh-board (26%) was achieved due to the formation of multi blisters affected by the too short pressing time at the current temperature. The best WA values were achieved at the highest pressing temperature/time for both Uso- and Wh-boards. The increase of density from 1000 to 1200 kg m^−3^ resulted in a significant decrease of the lowest WA value by 51% and 56% for Uso- and Wh-boards, respectively.

The significant linear models were suggested to describe WA depending on the variables for both Uso- and Wh-1200 boards:

WA_Uso1200_ = 46.09 − 0.15(T) − 0.33(t) (26)

WA_Wh1200_ = 35.78 − 0.04(T) − 0.79(t) (27)

Both variable factors were found to be significant (Table 4), describing the WA variation for the Uso-board in Equation (26) and only one significant factor t for the Wh-1200 board in Equation (27). In the case of the Uso-1200 board, increasing T by 1 °C and t by 1 min, WA will decrease by 0.15% and 0.33%, respectively. In turn, in the case of Wh-boards, increasing t by 1 min, WA will decrease by 0.79%.

The TS values of Uso- and Wh-1200 boards also decrease with increasing pressing variables, from 9% to 5% and from 11% to 6%, respectively (Figure 14). The obtained TS values at all pressing conditions are significantly lower for the Uso-board compared to the Wh-board. This indicates and proves that the binder-less board produced from SE Uso shives is more form-stable than that from SE wheat straw. From another point of view, all the obtained TS values from both board crops fully meet the standard requirement (≤20%) of EN 312 P3. The increase of density from 1000 to 1200 kg m^−3^, again, did not promote the improvement of TS values, even the increment by 33% in the case of the Wh-1200 board. This again proved some advantage of hemp shives vs. wheat straw in terms of the form stability of binder-less boards.

As in the case of WA, the significant linear models were suggested to describe also TS depending on the variables for both Uso- and Wh-1200 boards:

TS_Uso1200_ = 18.48 − 0.06(T) − 0.12(t) (28)

TS_Wh1200_ = 24.6 − 0.07(T) − 0.34(t) (29)

Both variable factors were found to be significant (Table 4), describing the TS variation for the Uso-board in Equation (28) and only one significant factor *t* for the Wh-1200 board in Equation (29). In the case of Uso-1200 boards, increasing *T* by 1 °C and *t* by 1 min, TS will decrease by 0.06% and 0.12%, respectively. In turn, in the case of Wh-1200 boards, increasing *t* by 1 min, TS will decrease by 0.34%.

#### 3.3.4. Model Fit

**Figure 15:**
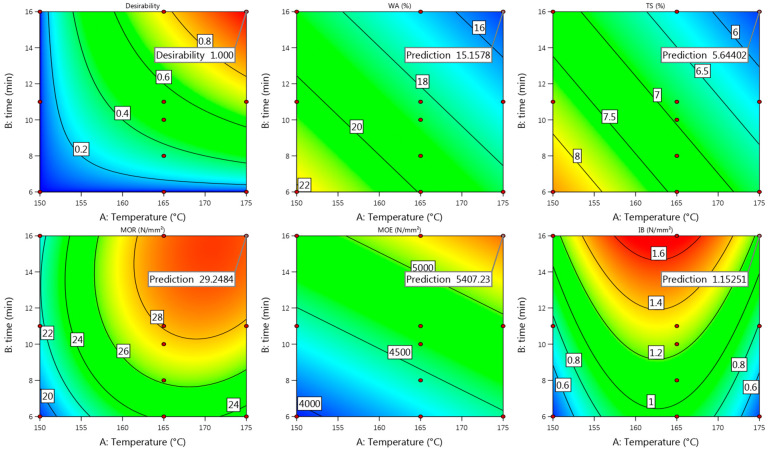
Contour plots with predicted values of Uso-1200 binder-less board properties at T = 175 °C and t = 16 min.

Summarizing all the obtained properties of Uso-1200 boards at each pressing condition, the best actual values were achieved at 175/16 (Figure 12, Figure 13 and Figure 14) and fit well with the predicted ones (Figure 15). As the predicted values of Uso-1200 board properties fit with the actual values within the confidence level of 95%, it proves the suitability of the obtained regression models proposed in Equations (20), (22), (24), (26) and (28).

**Figure 16:**
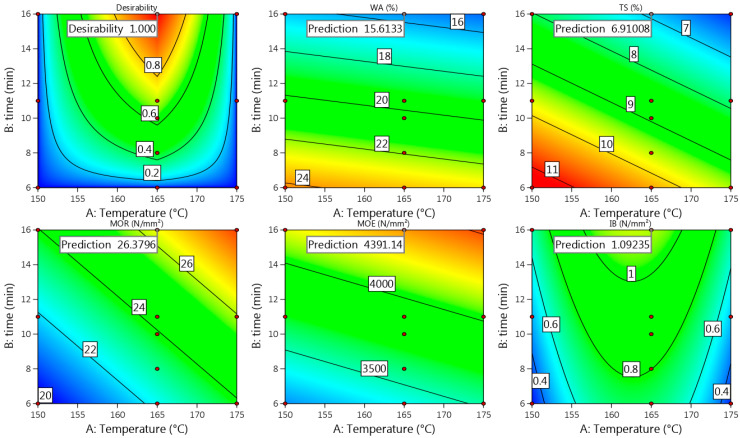
Contour plots with predicted values of Wh-1200 binder-less board properties at T = 165 °C and t = 16 min.

Comparing the achieved optimal actual values of the Wh-1200 board properties obtained at the pressing temperature of 165 °C and time of 16 min (Figure 12, Figure 13 and Figure 14) with the predicted ones (Figure 16), it could be concluded that those fit well, proving the suitability of the obtained models proposed in Equations (21), (23), (25), (27) and (29). In these conditions (165/16), even the insignificant regression models obtained for IB and MOE proposed in Equations (21) and (25) fit the predicted values within the confidence level of 95%.

Both Uso- and Wh-1200 boards, in terms of the obtained optimal values, fully meet the requirements of the standard EN 312 P3. Due to the high density, the obtained binder-less boards can be compared to the conventional hardboard restricted by the standard requirements of EN 625-2 HB (IB ≥ 0.5 N mm^−2^, MOR ≥ 25 N mm^−2^, TS ≤ 25%), which were successfully fulfilled not only at optimal conditions (Figure 12, Figure 13 and Figure 14).

### 3.4. Evaluation of Binder-Less Boards by FTIR Spectroscopy

**Figure 17:**
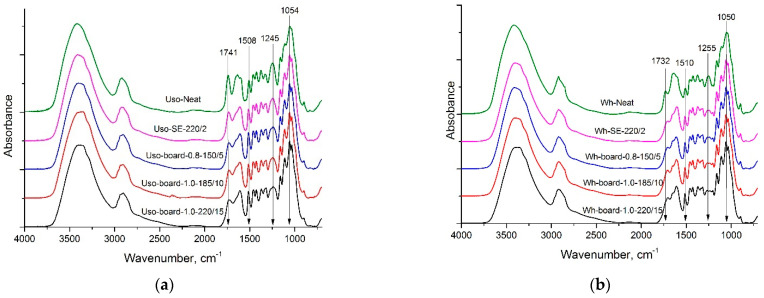
FTIR spectroscopy of neat, pre-treated and board samples from (**a**) Uso hemp shives and (**b**) wheat straw.

Fourier-transform infrared spectroscopy (FTIR) was used for analyses of the chemical composition changes of raw and pre-treated crops and the obtained boards. The chemical composition of wheat straw and hemp shives consists mainly of cellulose, hemicelluloses, and lignin in different proportion and various minor compounds; therefore, FTIR spectra show a similarity of the main absorption bands but differ in their intensity. The results of FTIR spectroscopy are presented in Figure 17. The absorption peaks maxima were assigned with the previously published literature data [36,37]. The neat crops represent typical FTIR spectra of lignocellulosic biomass with the following absorption peak maxima: a broad absorption peak around 3400 cm^−1^ for OH groups, an aliphatic (–CH_2_–) stretch region 2940–2840 cm^−1^, various carbonyl (C = O) regions between 1800 and 1600 cm^−1^, including the adsorption peak maximum around 1740 cm^−1^ for unconjugated C = O of the aliphatic ester group, for example, the acetyl group, aromatic skeletal vibration around 1510 cm^−1^ typical of lignin and a complex band of C–O–C, C–O, C–C stretching and C–OH bending around 1050 cm^−1^, more typical of carbohydrates. A comparison of the FTIR spectra of raw crops indicates that wheat straw contains more carbohydrates and less lignin than hemp shives, but hemp shives contain more esters (e.g., esterified hemicelluloses).

After SE pre-treatment, the broad absorption peak around 3400 cm^−1^ of OH groups remains, indicating a possible formation of hydrogen bonds during the hot-pressing of binder-less board, which enhance its IB [38]. The absorption peak maxima at 1741 cm^−1^ and around 1250 cm^−1^ (C–O stretch of acetate groups) drastically decreased. The peak ratio of carbohydrates (1054 cm^−1^) and lignin (1508 cm^−1^) absorption maxima decreased. This confirms the destruction and deacetylation of hemicelluloses by SE pre-treatment. Furthermore, the higher intensity of the absorption peaks maxima of carbohydrates and lignin is observed for all Uso samples compared to wheat samples indicating a higher content of hemicelluloses and lignin that is in accordance with the previously detected chemical composition [24]. In turn, this may lead to the explanation of obtained higher MOE of Uso boards mentioned in the Section 3.3.

The frequency of the carbonyl group absorption is determined by the structure of the rest molecule. Frequency shift away from its position have been considered by numerous studies. C = O stretching vibration of saturated carbonyl group is 1725–1705 cm^−1^ (ketone). Conjugation of a carbonyl group with unsaturated linkages (e.g., aryl or quinones) results in a lowering of absorption wavenumber (<1690 cm^−1^). Observed shift of carbonyl group absorption in the 1800–1600 cm^−1^ region to the smaller wavelength region could be explained by C = O groups conjugation, probably as a result of condensation reactions at thermal treatment [39]. Pressing at temperatures up to 185 °C did not significantly affect the chemical composition of boards from both crops. Increasing pressing temperature to 220 °C led to further hemicellulose destruction and deacetylation. It coincides with our previous observation that deacetylation at temperatures <200 °C does not occur [40].

## 4. Conclusions

Optimal hot-pressing conditions of binder-less board formation from steam-exploded (220 °C/2 min, logR0 = 3.83) hemp shives and wheat straw are reported. Based on the experimental design, which includes two factor variables of temperature (150–220 °C) and time (5–15 min) at three board density levels (800–1000–1200 kg m^−3^), statistically significant mathematical models were obtained to express the properties of the obtained boards. It is possible to obtain a binder-less board from both crops at different density levels; however, its quality is highly dependent on the pressing temperature and time. The optimal pressing temperature decreases with increasing density. The detected optimal conditions differ depending on the crop and density levels: for Uso- and Wh-800 boards—220 °C/10 min and 15 min, respectively; for Uso-1000 board—210 °C/10 min; for Wh-1000 board—185 °C/10 min; for Uso-1200 board—175 °C/16 min; for Wh-1200 board—165 °C/16 min. Despite the obtained good properties of the Wh-1200 board (IB = 1.45 N mm^−2^, MOR = 27.5 N mm^−2^, MOE = 4260 N mm^−2^, TS = 6%), SE wheat straw is not suggested for binder-less board production at this density level since blistering formation was not avoided at T ≥ 165 °C. The obtained binder-less boards from both crops at a density level of ≥ 1000 kg m^−3^ meet the requirements of the conventional particleboard Type P3 according to EN 312 suitable for application in humid condition. At the density level of 800 kg m^−3^, only the boards from SE wheat straw meet the standard values (IB = 0.64 N mm^−2^, MOE = 2750 N mm^−2^, MOR = 15.5 N mm^−2^, WA = 53%, TS = 4%), while Uso-800 boards do not meet the standard requirements due to a lower MOR value (IB = 0.64 N mm^−2^, MOE = 2950 N mm^−2^, MOR = 12 N mm^−2^, WA = 53%, TS = 5%). FTIR analysis testified that only the pressing temperature of 220 °C led to the further destruction and deacetylation of hemicelluloses.

## Figures and Tables

**Table 1:** Experimental design for the binder-less board pressing cycle.

Experimental No.	Designation	Temperature (T) °C	Time (t) min	Max Pressure (p) MPa
**Density: 800 kg m^−3^ and 1000 kg m^−3^**				
1	150/5	150	5	4.5
2	220/5	220	5	
3	150/15	150	15	
4	220/15	220	15	
5	150/10	150	10	
6	220/10	220	10	
7	185/5	185	5	
8	185/15	185	15	
9–11	185/10	185	10	
**Density: 1200 kg m^−3^**				
1	150/6	150	6	6
2	175/6	175	6	
3	150/16	150	16	
4	175/16	175	16	
5	150/11	150	11	
6	175/11	175	11	
7	163/6	162.5	6	
8	163/16	162.5	16	
9–11	163/11	162.5	11	

**Table 2:** Fit summary of the suggested models for each property of the boards with the density of 800 kg m^−3^.

Response	Suggested Model	Sequential *p*-Value	Lack of Fit *p*-Value	Adjusted R^2^	Predicted R^2^	Significant Factors *
**Uso-800**						
IB	Mean	<0.0001				- **
MOR	Linear	<0.0001	0.3385	0.9504	0.9190	A
MOE	Linear	0.0004	0.1216	0.8267	0.6929	A
WA	Quadratic	0.0001	0.7393	0.9906	0.9749	A, B, A^2^
TS	Linear	<0.0001	0.5365	0.9408	0.9021	A, B
**Wh-800**						
IB	Linear	0.0825	0.6715	0.3301	0.0216	-
MOR	Linear	<0.0001	0.2771	0.9273	0.8770	A
MOE	Quadratic	0.0439	0.0392	0.9755	0.8775	A, B, AB, B^2^
WA	Linear	0.0094	0.5385	0.6111	0.3534	A
TS	Quadratic	0.0241	0.2763	0.9922	0.9692	A, B, AB, A^2^

* Pressing factors with a *p*-value < 0.05 in the suggested model: A—temperature, B—time. ** No significant factor was found by the ANOVA of the suggested model.

**Table 3:** Fit summary of suggested models for each property of the boards with the density of 1000 kg m^−3^.

Response	Suggested Model	Sequential *p*-Value	Lack of Fit *p*-Value	Adjusted R^2^	Predicted R^2^	Significant Factors *
**Uso-1000**						
IB	Quadratic	0.1513	0.8455	0.1054	-0.9481	- **
MOR	2FI	0.0339	0.0914	0.8296	0.6137	A, AB
MOE	Quadratic	0.0070	0.3781	0.9136	0.6655	A, AB, A^2^
WA	Quadratic	0.0030	0.5938	0.9266	0.7669	A, B, AB, A^2^
TS	Linear	<0.0001	0.1875	0.9590	0.9243	A, B
**Wh-1000**						
IB	Quadratic	0.0107	0.5748	0.9021	0.6675	A, B, A^2^
MOR	Quadratic	0.0051	0.1817	0.8331	0.4520	A, A^2^
MOE	Quadratic	0.0020	0.0354	0.8799	0.5662	A, B, A^2^
WA	Quadratic	0.0050	0.9284	0.8863	0.8110	B, AB, A^2^, B^2^
TS	Linear	<0.0001	0.0234	0.9010	0.8052	A, B

* Pressing factors with a *p*-value < 0.05 in the suggested model: A—temperature, B—time. ** No significant factor was found by the ANOVA of the suggested model.

**Table 4:** Fit summary of the suggested models for each property of the boards with the density of 1200 kg m^−3^.

Response	Suggested Model	Sequential *p*-Value	Lack of Fit *p*-Value	Adjusted R^2^	Predicted R^2^	Significant Factors *
**Uso-1200**						
IB	Quadratic	0.0382	0.8367	0.7026	0.3376	B, A^2^
MOR	Quadratic	0.0327	0.8499	0.8350	0.6441	A, B, A^2^
MOE	Linear	0.0159	0.7065	0.5560	0.2768	B
WA	Linear	0.0091	0.0287	0.6143	0.3394	A, B
TS	Linear	0.0008	0.1690	0.7867	0.6487	A, B
**Wh-1200**						
IB	Quadratic	0.0965	0.4493	0.3582	−0.6734	A^2^
MOR	Linear	0.0258	0.2338	0.4992	0.0302	A, B
MOE	Linear	0.0554	0.8338	0.3937	0.1575	B
WA	Linear	0.0007	0.2946	0.7971	0.6726	B
TS	Linear	0.0160	0.7223	0.5555	0.3833	B

* Pressing factors with a *p*-value < 0.05 in the suggested model: A—temperature, B—time.

## Data Availability

Not applicable.
